# TYRO3 as a molecular target for growth inhibition and apoptosis induction in bladder cancer

**DOI:** 10.1038/s41416-019-0397-6

**Published:** 2019-02-15

**Authors:** Florent Dufour, Linda Silina, Hélène Neyret-Kahn, Aura Moreno-Vega, Clémentine Krucker, Narjesse Karboul, Marion Dorland-Galliot, Pascale Maillé, Elodie Chapeaublanc, Yves Allory, Nicolas Stransky, Hélène Haegel, Thierry Menguy, Vanessa Duong, François Radvanyi, Isabelle Bernard-Pierrot

**Affiliations:** 1 Institut Curie, PSL Research University, CNRS, UMR144, Equipe Labellisée Ligue contre le Cancer, 75005 Paris, France; 2 Sorbonne Universités, UPMC Université Paris 06, CNRS, UMR144, 75005 Paris, France; 3 AP-HP, Henri-Mondor Hospital, Department of Pathology, 94000 Créteil, France; 4 Institut Curie, Department of Pathology, 92210 Saint-Cloud, France; 5 ELSALYS BIOTECH SAS, 69007 Lyon, France

**Keywords:** Bladder cancer, Targeted therapies

## Abstract

**Background:**

Muscle-invasive bladder cancer (MIBC) is an aggressive neoplasm with poor prognosis, lacking effective therapeutic targets. Oncogenic dependency on members of the TAM tyrosine kinase receptor family (TYRO3, AXL, MERTK) has been reported in several cancer types, but their role in bladder cancer has never been explored.

**Methods:**

TAM receptor expression was evaluated in two series of human bladder tumours by gene expression (TCGA and CIT series), immunohistochemistry and western blotting analyses (CIT series). The role of the different TAM receptors was assessed by loss-of-function experiments and pharmaceutical inhibition in vitro and in vivo.

**Results:**

We reported a significantly higher expression of TYRO3, but not AXL or MERTK, in both non-MIBCs and MIBCs, compared to normal urothelium. Loss-of-function experiments identified a TYRO3-dependency of bladder carcinoma-derived cells both in vitro and in a mouse xenograft model, whereas AXL and MERTK depletion had only a minor impact on cell viability. Accordingly, TYRO3-dependent bladder tumour cells were sensitive to pharmacological treatment with two pan-TAM inhibitors. Finally, growth inhibition upon TYRO3 depletion relies on cell cycle inhibition and apoptosis associated with induction of tumour-suppressive signals.

**Conclusions:**

Our results provide a preclinical proof of concept for TYRO3 as a potential therapeutic target in bladder cancer.

## Background

Bladder cancer is the ninth most common cancer worldwide, with ~430,000 new cases diagnosed in 2012 and 165,000 deaths annually.^1^ Muscle-invasive bladder cancers (MIBCs) account for 25% of bladder tumours at initial diagnosis and are life-threatening. Indeed, despite radical cystectomy with cisplatin-based neoadjuvant and/or adjuvant chemotherapy, the standard treatment for MIBC, overall survival is only about 50% at 5 years, and is as low as 5% in cases of distant metastasis. Immune checkpoint inhibitors can be beneficial in patients with bladder cancer,^2^ but they are effective in only a limited number of patients (20%). New effective therapies targeting both MIBCs and non-muscle-invasive tumours (NMIBCs), which often recur, are therefore required. Efforts to decipher the molecular mechanisms involved in bladder carcinogenesis have led to the identification of a number of possible new treatment targets, such as mTOR in patients with *TSC1* mutations, epidermal growth factor receptor 2 (HER2)/ERBB2 in HER2-positive tumours, EGFR in basal-like tumours, and fibroblast growth factor receptors, particularly in patients harbouring mutations or gene fusions of *FGFR3*.^3–5^

The TAM family of receptor tyrosine kinases (RTKs), which includes TYRO3, AXL and MERTK, has emerged as new therapeutic target in many types of cancer, but their role in bladder cancer has not yet been determined. TAM receptors can be activated by the vitamin K-dependent protein GAS6 (growth arrest-specific 6), but ligand-independent activation has also been described.^6^ TAM receptors are involved in immune control, inflammation and homeostasis in several organs, including the nervous, reproductive and vascular systems.^7,8^ Aberrant TAM receptor expression has been reported in diverse solid and hematopoietic tumours in humans, with effects on apoptosis, growth, metastasis and chemosensitivity, although the underlying mechanisms remain incompletely understood.^8–10^ AXL has been, so far, more extensively studied in cancers than either MERTK or TYRO3.

In this study, we investigated TAM receptor's expression in two independent cohorts of bladder tumours and evaluated the influence of each receptor on the regulation of growth/survival in urothelial bladder cancer-derived cell lines (UBC cell lines).

## Materials and methods

### Cell lines

The human bladder cancer-derived cell lines RT112, UM-UC-5, UM-UC-9, VM-CUB-1, 5637, 647 V and HT1376 were obtained from DSMZ (Heidelberg, Germany). MGH-U3 cells were kindly provided by Dr. Francisco X. Real (CNIO, Madrid). MGH-U3, UM-UC-5, UM-UC-9, VM-CUB-1, 647 V and HT1376 cells were cultured in DMEM, whereas RT112, and 5637, cells were cultured in RPMI. Media were supplemented with 10% foetal bovine serum (FBS) (ThermoFisher Scientific, Courtaboeuf, France). Cells were kept at 37 °C, under an atmosphere containing 5% CO_2_. The identity of the cell lines used was checked by analysing genomic alterations on comparative genomic hybridization arrays (CGH array) and sequencing genes known to be mutated: *RAS, TP53, FGFR3* and *PIK3CA*. The cells were routinely checked for mycoplasma contamination.

### Human bladder samples

We assessed expression of the *TYRO3, AXL*, *MERTK and GAS6* genes by RT-qPCR, using 169 bladder tumour samples (87 NMIBCs and 82 MIBCs) from the previously described CIT-series cohort (“Carte d’Identité des Tumeurs” or “Tumour identity card”) of bladder tumours.^5,11^ Seven normal urothelial samples were obtained from fresh urothelial cells scraped from the normal bladder wall and dissected from the lamina propria during organ procurement from a cadaveric donor for transplantation. RNA, DNA and protein were extracted from the surgical samples by cesium chloride density centrifugation, as previously described.^5,12^ We used protein extracted from 21 human bladder tumours from the CIT-series (4 NMIBCs and 17 MIBCs) for western blot analysis.^5,12^ Lyophilized proteins were solubilised in 1X Laemmli sample buffer and boiled for 10 min. Protein concentrations were determined with the BioRad Bradford Protein Assay Kit (BioRad, Marnes-la-Coquette, France) and TAM protein levels were assessed by immunoblotting.

### RNA extraction and real-time reverse transcription-quantitative PCR

RNA was isolated from cell lines and xenografts with RNeasy Mini kit (Qiagen, Courtaboeuf, France). Reverse transcription was performed with 1 µg of total RNA, and a high-capacity cDNA reverse transcription kit (ThermoFisher Scientific). A predesigned assay was used to quantify expression of the TATA-box binding protein (*TBP*) reference gene (ThermoFisher Scientific, Ref: 4326322E). Custom-designed assays were used to measure expression of the *TYRO3, AXL*, *MERTK* and *GAS6* genes. Primers and probes were designed with Probe Finder software at the Universal Probe Library Assay Design Center (Roche). RT-qPCR settings were as described elsewhere.^5^ For each gene of interest, the amount of mRNA was normalised against the *TBP* reference gene by the 2^-ΔΔCt^ method.

TYRO3 (Roche Universal Probe Library probe ID: 14):

5′- GAGGATGGGGGTGAAACC-3′ (sense strand)

5′- ACTGTGAAAAATGGCACACCT-3′ (antisense strand)

AXL (Roche Universal Probe Library probe ID: 76):

5′-AACCAGGACGACTCCATCC-3′ (sense strand)

5′-AGCTCTGACCTCGTGCAGAT-3′ (antisense strand)

MERTK (Roche Universal Probe Library probe ID: 6):

5′-ATTGGAGACAGGACCAAAGC-3′ (sense strand)

5′-GGGCAATATCCACCATGAAC-3′ (antisense strand)

GAS6 (Roche Universal Probe Library probe ID: 17):

5′-ATGGCATGTGGCAGACAAT-3′ (sense strand)

5′-CCCTGTTGACCTTGATGACC-3′ (antisense strand)

### Immunohistochemistry

Formalin-fixed, paraffin-embedded 3 µm tissue sections of tumours from the CIT-series were placed on poly-L-lysine coated slides. The paraffin was removed by immersion in xylene and the section was rehydrated by immersion in a graded series of alcohol concentrations. Antigens were retrieved by heating sections at 95 °C in 10 mM citrate buffer pH 9 (Microm Microtech France, Brignais, France) for 20 min. Endogenous peroxidase activity was inhibited by incubation in 3% H_2_O_2_. The sections were then incubated in Quanto Protein Block solution (Microm Microtech France) for 1 h to minimise nonspecific staining. The sections were then incubated with a rabbit polyclonal anti-TYRO3 antibody (Ref: HPA071245, Sigma-Aldrich, Saint-Quentin Fallavier, France) diluted 1:50 in antibody diluent solution (Diamond antibody diluent, Cell Marque, Rocklin, USA) for 1 h at 37 °C. Antibody binding was detected with N-Histofin® Simple staining and a DAB detection kit (Microm Microtech, France), according to the manufacturer’s instructions. Antibody specificity for TYRO3 was assessed with formalin-fixed paraffin-embedded 5637 bladder cancer cells, which express TYRO3, AXL and MERTK, after transfection with either control siRNA or *TYRO3* siRNA#2.

### Flow cytometry

Bladder cancer cells (1 × 10^6^ cells/condition) were collected with Accutase (Sigma-Aldrich), washed in cold phosphate-buffered saline (PBS), and incubated with 1 µg of anti-TYRO3 clone 5B4 (Ref: GTX83459, GeneTex, Irvine, USA) or isotype control (Ref: MAB003 R&D Systems, Lille, France) antibody in PBS supplemented with 0.5% bovine serum albumin (BSA) for 45 min on ice. The cells were then washed three times in cold 0.5% BSA PBS and incubated for 30 min on ice with 2 μg/ml phycoerythrin-conjugated goat-anti-mouse Ig (Ref: 550589, BD Biosciences, Le Pont de Claix, France) in 0.5% BSA PBS. Cells were washed three times in 0.5% BSA PBS and the fluorescence signal was acquired with a BD LSR II flow cytometer (BD Biosciences).

### Cell viability after transfection with siRNA and treatment with small compounds

For siRNA transfection, cells were transfected with either 20 nM (MGH-U3, RT112, UM-UC-5, UM-UC-9, 5637 and 647 V) or 5 nM (VM-CUB-1) siRNA in the presence of Lipofectamine RNAi Max reagent (ThermoFisher Scientific), in accordance with the manufacturer’s protocol. siRNAs were purchased from Ambion and Qiagen. For the control siRNA, we used a Qiagen control targeting luciferase (Ref: SI03650353). The sequences of the siRNAs are described in Table S1.

For small-compound inhibitor treatments, MGH-U3, RT112, UM-UC-5, UM-UC-9 and VM-CUB-1 cells were used to seed 96-well plates with appropriate culture media supplemented with 10% FBS. After 24 h, the cells were treated with either UNC-2025 (Ref. S7576, Euromedex, Souffelweyersheim, France) or BMS-777607 (Ref. HY-12076, CliniSciences, Nanterre, France).

Cell viability was assessed with the CellTiter-Glo assay (Promega, Charbonnières-les-Bains, France).

### Protein extraction and immunoblotting

Cell lysates were prepared and immunoblotting was carried out as previously described.^13^ Anti-TYRO3 (Ref. 5585), AXL (Ref. 8661), MERTK (Ref. 4319), caspase-8 (Ref. 9746), cleaved caspase-3 (Ref. 9664), cleaved PARP (Ref. 5625), FOXM1 (Ref. 5436), pRB (Ref. 9309), phospho-pRB Ser780 (Ref. 9307), CYCLIN D1 (Ref. 2926), AURORA A (Ref. 14475), AURORA B (Ref. 3094), SURVIVIN (Ref. 2808), c-MYC (Ref. 9402), anti-mouse IgG, HRP-linked (Ref. 7076) and anti-rabbit IgG, HRP-linked (Ref. 7074) antibodies were purchased from Cell Signaling Technology (Ozyme, Montigny-le-Bretonneux, France). Anti-α-TUBULIN (Ref. T6199) and anti-β-ACTIN (Ref. A2228) antibodies were obtained from Sigma-Aldrich. For peptide-N-glycosidase F (PNGaseF), we digested 10 µg of protein with PNGaseF, according to the manufacturer’s instructions (New England Biolabs, Evry, France).

### Soft agar assay

MGH-U3 and RT112 cells, untransfected or transfected with siRNA, were used to seed 12-well plates containing DMEM supplemented with 10% FBS and 1% agar, in triplicate (20,000 cells/well). The medium was changed weekly. The plates were incubated for 14 days and colonies larger than 50 µm in diameter, as measured with a phase-contrast microscope equipped with a measuring grid, were counted.

### DNA array

For the identification of genes displaying changes in expression after *TYRO3* knockdown in MGH-U3, RT112 and UM-UC-5 cells, we transfected the cells for 40 h with *TYRO3* siRNA#1, *TYRO3* siRNA#2 or *TYRO3* siRNA#3. mRNA was extracted and purified with RNeasy Mini kit (Qiagen). Total RNA (200 ng) from control and siRNA-treated MGH-U3, RT112 and UM-UC-5 cells was analysed with the Affymetrix U133 Plus 2.0 DNA array. Raw gene expression data were normalised and summarised by the RMA (robust multi-array averaging) method (R package affy) with a customised chip definition developed by Microarray Lab, BrainArray (HGU133Plus2_Hs_ENTREZG_v18).^14,15^ One log2-transformed signal value per gene was obtained. The microarray data described here are available from GEO (https://www.ncbi.nlm.nih.gov/geo/) under accession number GSE100025. The LIMMA algorithm was used to identify genes differentially expressed between *TYRO3* siRNA-treated (*n* = 9) and lipofectamine-treated control (*n* = 9) cells.^16^ The *p* values were adjusted for multiple testing by the Benjamini–Hochberg FDR procedure. Genes with |log_2_FC| ≧ 0.58, and with a FDR below 5% were considered to be differentially expressed.

### Xenograft models

Six-week-old female Swiss *nu/nu* mice (Charles River Laboratories, Saint-Germain-Nuelles, France) were raised in the animal facilities of Institut Curie, in specific pathogen-free conditions. They were housed and cared for in accordance with the institutional guidelines of the French National Ethics Committee (*Ministère de l’Agriculture et de la Forêt, Direction de la Santé et de la Protection Animale*, Paris, France), under the supervision of authorised investigators. Mice received a subcutaneous injection, into each flank (dorsal region), of 5 × 10^6^ MGH-U3 bladder cancer cells in 100 µl PBS. For each study, with each of the cell lines, mice were randomly separated into groups of six mice when tumours reached a volume of 50 mm^3^ (±5). The mice bearing MGH-U3 xenografts were treated three times weekly, for three weeks, with an intraperitoneal injection of 4 µg siRNA (*TYRO3* siRNA#1 or corresponding scramble siRNA) or of vehicle (PBS). Tumour size was measured twice weekly with a calliper, and tumour volume in mm^3^ was calculated as follows: *π*/6 × (largest diameter) × (shortest diameter)^2^. The tumours were removed at the end of treatment. Part of the tumour was flash-frozen in liquid nitrogen for mRNA extraction. The rest was fixed in 4% formol and embedded in paraffin.

### TUNEL assay

DNA fragmentation was evaluated with a TUNEL (deoxynucleotidyl transferase (Tdt)-mediated nick-end labelling) assay detection kit (Sigma-Aldrich), according to the manufacturer’s instructions.

### Statistical analysis

TAM expression in tumours was analysed with Mann–Whitney test. Linear Models for Microarray Data (LIMMA)^16^ was used to analyse DNA array experiments involving simultaneous comparisons between large numbers of RNA targets. All functional experiments were carried out twice or three times, in triplicate. To compare data between cell lines, two-tailed *t*-tests were used. The control siRNA (Luciferase GL2 siRNA) or DMSO group was used as the reference group. Data are expressed as means ± SD, and differences with a *p* value < 0.05 were considered statistically significant. For the comparison of data between xenograft treatments, the non-parametric Mann–Whitney test was used, with the vehicle group as the reference group. All statistical analyses were performed with Prism7.0b (GraphPad Software, Inc).

## Results

### TYRO3 is highly expressed in bladder carcinomas

**Fig. 1:**
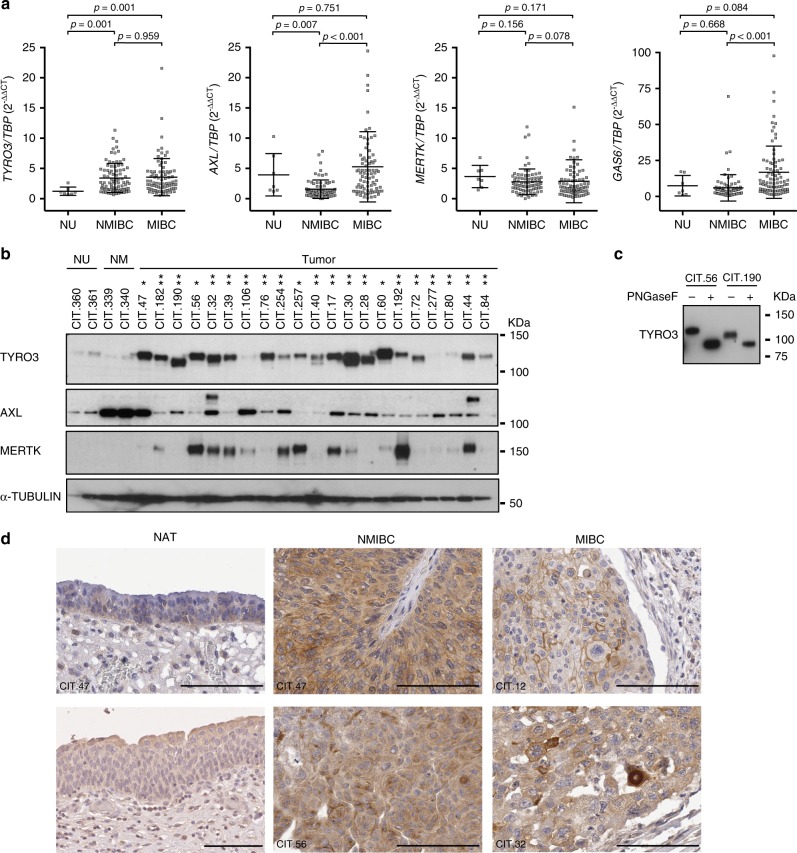
TYRO3 is highly expressed in bladder cancer and is N-glycosylated. **a** *TYRO3, AXL, MERTK* and *GAS6* mRNA levels, as measured by RT-qPCR, in the CIT cohort of non-muscle-invasive bladder cancers (NMIBCs, *n* = 87) and muscle-invasive bladder cancers (MIBCs, *n* = 82) and in normal urothelium (NU, *n* = 7). The significance of differences was assessed by Mann–Whitney test, and the means and standard errors are shown. **b** Lysates from a subset of human tumours from the CIT cohort and from normal urothelium (NU) and normal vesical muscle (NM) were analysed by western blotting with antibodies targeting the indicated proteins. NMIBCs and MIBCs were identified by one and two asterisks, respectively. **c** TYRO3 immunoblots for tumour lysates (CIT.56 and CIT.190) treated with peptide-N-glycosidase F (PNGaseF). **d** Representative images of TYRO3 immuno-histochemical staining for a panel of human bladder tumours (NMIBC and MIBC) and urothelium from histologically normal tissue adjacent to the tumour (NAT). Black bars represent 100 μm. IHC validation experiments are available in supplementary figure S3

We first investigated the role of TAM receptors in human bladder carcinoma, by analysing, using RT-qPCR, the levels of expression of *AXL*, *MERTK*, *TYRO3* and *GAS6* in our previously described CIT cohort of 169 bladder tumours encompassing 87 NMIBCs and 82 MIBCs.^5,11^ Seven normal urothelium samples were used as controls (Fig. 1a). Expression levels for the three receptors and their ligand were heterogeneous in tumours, with only *TYRO3* significantly elevated in NMIBCs and MIBCs relative to normal samples. *GAS6* was more strongly expressed in MIBCs than in NMIBCs, suggesting that GAS6 might increase the autocrine or paracrine activation of TYRO3 in muscle-invasive tumours (Fig. 1a). Higher *TYRO3* expression in MIBCs relative to normal samples was confirmed by an analysis of publicly available RNA-seq data from the TCGA cohort for 405 MIBCs (primary tumours only) and 14 normal samples^17^ (Supplementary Figure S1). As in the CIT cohort, no increased expression of *AXL*, *MERTK* or *GAS6* was observed in MIBCs compared to normal samples but surprisingly in this data set a decreas of *GAS6* and *AXL* mRNA levels could be observed. This discrepancy could be due to the difference of so-called normal samples in the two series: pure normal urothelium samples in the CIT cohort, versus peritumoural bladder tissue with a normal histological appearance in the TCGA cohort. The difference in the number of samples considered could also influence the results. We further analysed TAM receptor expression at the protein level, by western blots of a subset of tumours from our CIT cohort (Fig. 1b and Supplementary Figure S2). Consistent with the results of mRNA analysis, TYRO3 levels in almost all tumours were higher than those in normal urothelium and vesical muscle samples, whereas AXL levels were higher than those in the urothelium in only a few tumours and this protein was abundant in the vesical muscle. However, strong MERTK expression was surprisingly detected in 8 of 21 tumours. Different migration profiles in immunoblotting assays were observed for TYRO3, possibly due to post-translational modifications of the receptor (Fig. 1b). An analysis of the TYRO3 sequence with NetNGlyc1.0 software identified seven potent N-glycosylation sites (not shown). The treatment of two tumour protein samples with peptide N-glycanase F (PNGaseF), an enzyme that removes N-linked glycans, shifted the TYRO3 band to the expected theoretical molecular weight of 96 kDa, confirming the N-glycosylation of this receptor (Fig. 1c). We examined the protein expression pattern of TYRO3 in clinical samples from the CIT cohort by immunohistochemistry using an anti-TYRO3 antibody. We had validated the specificity of the antibody using paraffin-embedded 5637 cells expressing the three TAM receptors and that were transfected or not with *TYRO3* siRNA (Supplementary Figure S3). No or weak TYRO3 staining was observed on urothelium from histologically normal tissue adjacent to the tumour (NAT), whereas TYRO3 was highly expressed by tumour epithelial cells and not by stromal cells in both NMIBCs and MIBCs (Fig. 1d). Strikingly, but as previously observed in colorectal tumour cells,^18^ TYRO3 staining was mainly detected in the cytoplasm of tumour cells and also at the plasma membrane. Overall, these results suggest that TYRO3 may play a role in bladder tumours.

### TAM receptors are expressed in bladder cancer cell lines, but TYRO3 is the main TAM involved in modulating cell viability

**Fig. 2:**
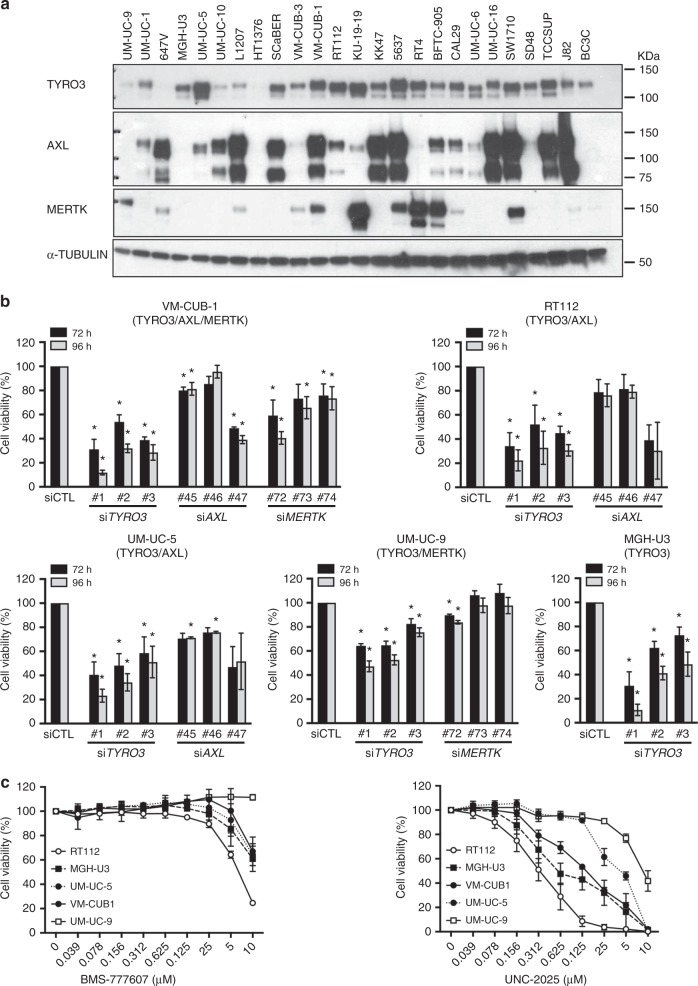
TAM knockdown and pharmaceutical inhibition studies in bladder cancer cell lines reveal the major contribution of TYRO3 to cell viability. **a** Lysates from a panel of human urothelial bladder cancer cell lines were analysed by western blotting for TAM expression. Alpha tubulin served as a loading control. **b** Knockdown of *TYRO3*, *AXL* and *MERTK* with three different siRNAs for each receptor, in a panel of bladder cancer cell lines. Cell survival was quantified by CellTiter-Glo at 72 h and 96 h. The TAM receptor(s) that is (are) expressed in each cell line is (are) indicated beneath their name. **c** Dose-response curves for bladder cancer cells treated with BMS-777607 and UNC-2025, two pan-TAM inhibitors. Cell viability was quantified by CellTiter-Glo at 72 h. The data shown are the means of three independent experiments, and error bars represent the SD. *p < 0.05. Statistical analyses based on unpaired *t*-tests with Welch’s correction are shown in supplementary tables S2 and S3

We then investigated the protein expression of the TAM receptors by western blot in a panel of cell lines derived from human urothelial bladder cancer (UBC) (Fig. 2a). Consistent with our results for human bladder tumours (Fig. 1b), TYRO3 was the TAM receptor most frequently expressed in UBC cell lines (in 21 out of 25 cell lines), followed by AXL (13/25) and MERTK (7/25). Most of the UBC cell lines strongly expressed at least two TAM receptors, but MGH-U3 and BC3C expressed only TYRO3 (Fig. 2a). We then used a loss-of-function approach based on siRNA to investigate whether TAM receptors were required for cell growth/survival (Fig. 2b). We investigated the potential redundancy and relative importance of the three receptors, by selecting one cell line expressing all three TAM receptors (VM-CUB-1), three cell lines expressing TYRO3 together with AXL or MERTK (RT112, UM-UC-5 and UM-UC-9) and one cell line expressing only TYRO3 (MGH-U3) (Fig. 2a, b). Western blot analysis showed that each targeted TAM was effectively silenced 72 h after transfection and that the knockdown of one TAM receptor had mostly no effect on the other TAM receptors with the exception of *TYRO3* knockdown using siRNA#1 that reduced AXL and increased MERTK expression, particularly in RT112 (Supplementary Figure S4). *TYRO3* knockdown significantly and strongly decreased cell viability in all the cell lines tested, regardless of the expression levels of other TAM receptors (by 60 to 75%, 96 h after transfection, depending on the cell line), demonstrating the high dependence for growth of bladder cancer cell lines on TYRO3 expression and the absence of redundancy with the other TAM receptors (Fig. 2b and Supplementary Table S2). Moreover, when TYRO3 was co-expressed with other TAM receptors, the effects of *TYRO3* silencing were consistently more important than those of *AXL* or *MERTK* knockdown, highlighting the predominant role of TYRO3 in the modulation of bladder cancer cell viability and suggesting the potential for therapeutic targeting of TYRO3 (Fig. 2b). This inhibition of TYRO3 could be achieved using specific blocking antibodies as flow cytometry experiments highlighted TYRO3 expression at the cell surface of the TYRO3-dependent cell lines (MGH-U3, UM-UC-5 and RT112 (Supplementary Figure S5)). However, as no such blocking antibodies were commercially available, we carried out TYRO3 inhibition experiments using two pan TAM receptor inhibitors, UNC-2025 (IC_50_ values are 18 nM, 7.5 nM and 0.7 nM for TYRO3, AXL and MERTK, respectively) and BMS-777607 (IC_50_ values are 4.3 nM, 1.1 nM and 14 nM for TYRO3, AXL and MERTK, respectively).^9,19,20^ These molecules are also described to inhibit with high affinity two other tyrosine kinase receptors, MET (IC_50_: 3.9 nM and 364 nM) and FLT3 (IC_50_; 16 nM and 0.35 nM); IC_50_ given for BMS-777607 and UNC-2025 respectively.^19,20^ Consistent with their affinity profile for the TAM receptors, treatment of VM-CUB-1 cells with 5 µM of BMS-777607 or UNC-2025 inhibited the phosphorylation of TYRO3, AXL and MERTK (Supplementary Figure S6). Pharmacological inhibition of TAM receptors with UNC-2025 and BMS-777607 significantly decreased in a dose-dependent manner the viability of RT112, MGH-U3, VM-CUB-1 and UM-UC-5 cells, whereas UM-UC-9 cells were more resistant (Fig. 2c and Supplementary Table S3). In line with our TAM receptor depletion experimental results (Fig. 2b), these effects appeared to be more strongly linked to TYRO3 inhibition. Indeed, MGH-U3 cells, which express only TYRO3, and RT112, which do not express MERTK and are resistant to *AXL* silencing, were not only the most sensitive cell lines to TYRO3 depletion but also to the TAM receptor inhibitors. Although we cannot rule out that a double or a triple knockdown could have a greater impact on cell viability than a single knockdown, we did not observe a greater sensitivity of VM-CUB-1, which expresses TYRO3, AXL and MERTK, to the pan-TAM inhibitors than the cell lines expressing TYRO3 only or in combination with a second TAM receptor. In the other hand, we could not exclude that MET or FLT3 inhibition also contributed to the observed phenotype since the cells express both receptors (data not shown).

### *TYRO3* knockdown reduces anchorage-independent cell growth in vitro and tumour growth in vivo

**Fig. 3:**
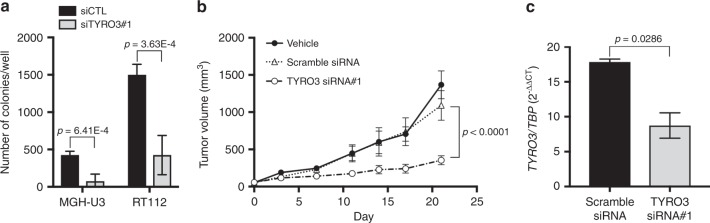
*TYRO3* depletion decreases colony-forming potential and tumourigenesis. **a** Impact of *TYRO3* knockdown on the anchorage-independent growth of MGH-U3 and RT112 cells. Colonies in soft agar with a diameter larger than 50 μm were quantified 14 days after transfection with a control siRNA (siCTL) or with *TYRO3* siRNA#1. Three experiments were performed, and the results shown are from a representative experiment conducted in triplicate. The data shown are means ± SD. Unpaired *t*-tests were used for statistical comparison. **b** MGH-U3 bladder cancer cells were injected subcutaneously into nude mice (*n* = 6 animals/group, two xenografts per animal (one in each flank)). When tumour volume reached 50( ± 5) mm^3^, tumour-bearing mice were treated three times weekly with intraperitoneal injections of 4 µg control scramble siRNA or *TYRO3* siRNA#1 (the first injection corresponds to day 0). Tumour sizes were measured at the indicated time points and tumour volumes were calculated. Data are presented as means ± SEM. Tumour volumes were compared by Wilcoxon’s test. **c** MGH-U3-derived xenograft tumours from mice treated with scramble siRNA or *TYRO3* siRNA#1 were lysed and *TYRO3* expression was measured by RT-qPCR. *TBP* was used as housekeeping gene. The data shown are means ± SD of two independent experiments performed in triplicate. Statistical analysis was performed with unpaired *t* tests

We then investigated whether this identified role of TYRO3 in bladder cancer cells was relevant to clonogenic cells, by analysing the consequences of *TYRO3* knockdown for the anchorage-independent growth of TYRO3-dependent bladder cancer cell lines in vitro and tumour growth in vivo. *TYRO3* depletion significantly decreased the number of viable MGH-U3 and RT112 colonies in soft agar assays, by 80 and 60% respectively, demonstrating a role for TYRO3 in regulating the survival of clonogenic cancer cells (Fig. 3a).

We also found that the treatment of established MGH-U3 xenografts in nude mice with intraperitoneal injections of *TYRO3* siRNA reduced significantly the tumour growth as compared to in similar tumours treated with a scramble siRNA (≈354 mm^3^ versus 1090 mm^3^ at day 21, *p* < 0.0001) (Fig. 3b). This inhibition of tumour growth was associated with significantly lower *TYRO3* mRNA levels on RT-qPCR (Fig. 3c).

These results indicate that the genetic depletion and pharmaceutical inhibition of TYRO3 lead to decreases in bladder cancer cell viability and tumour outgrowth ability, identifying TYRO3 as a potential therapeutic target.

### TYRO3 depletion induces tumour suppressor pathways and apoptosis, while inhibiting proliferative pathways

**Fig. 4:**
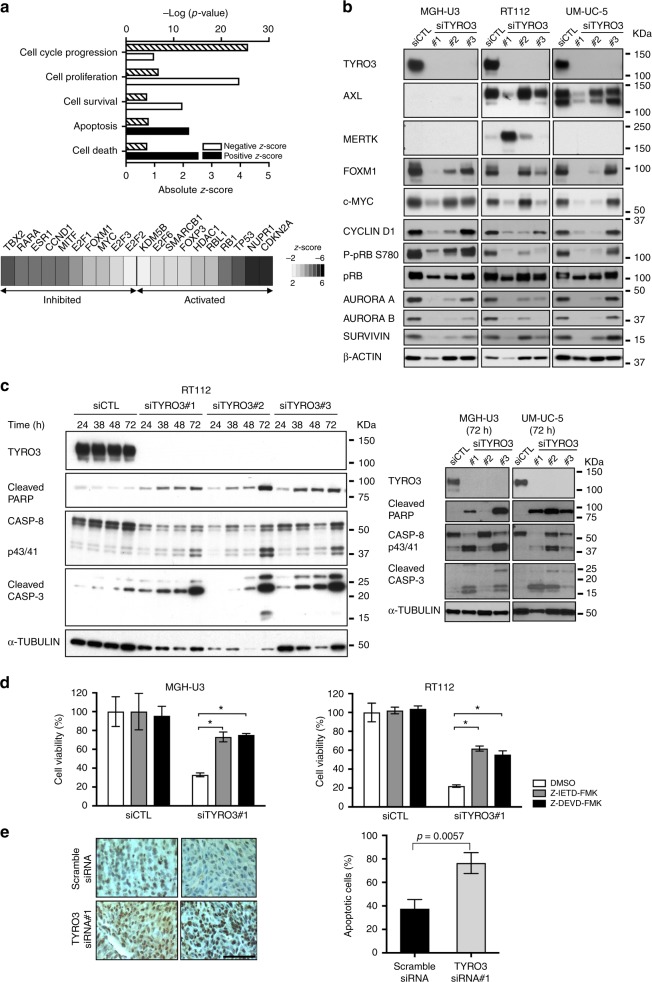
TYRO3 silencing inhibits the cell cycle and cell proliferation and induces apoptosis. **a** Transcriptomic analysis upon TYRO3 knockdown in MGH-U3, RT112 and UM-UC-5 cells. Ingenuity Pathway Analysis (IPA) was performed for a list of 284 genes differentially expressed upon TYRO3 silencing (fold-change |log2| ≥ 0.58 and a p value < 0.05). Upper panel: Histograms showing the cellular functions predicted by IPA to be deregulated after TYRO3 knockdown. *p* values (hatched bar), and negative (white bar) and positive (black bar) absolute z-scores are indicated. Lower panel: Diagram showing the upstream regulators (transcription factors) significantly predicted by IPA to be involved in the regulation of gene expression observed after TYRO3 knockdown (*p* value < 1E-6). **b**, **c**. Western blot analysis of TYRO3, AXL, MERTK, FOXM1, c-MYC, CYCLIN D1, phospho-pRB (P-pRB S780) and total pRB, AURORA A and B, SURVIVIN levels in total cell lysates from MGH-U3, RT112 and UM-UC-5 cells transfected with a control siRNA (siCTL) or with TYRO3 siRNAs (siTYRO3#1, siTYRO3#2 and siTYRO3#3) for 72 h. **c** The activation of apoptosis was assessed at the indicated period of time by monitoring cleaved PARP, caspase-8 (CASP-8) levels, with detection of the profrom (CASP-8) and cleaved form (p43/41), and caspase-3 (CASP-3). Western blot results are representative of at least two independent experiments. **d** MGH-U3 and RT112 cells were transfected with either a control siRNA (siCTL) or with TYRO3 siRNA#1 for 24 h. They were then treated with DMSO, 50 μM Z-IETD-FMK (caspase-8 inhibitor) and 50 μM Z-DEVD-FMK (caspase-3/7 inhibitor) for 48 h. Cell viability was assessed by measuring MTT incorporation at 72 h. The results are expressed as the means ± SD of three experiments. An unpaired *t*-test was used for statistical analysis. **e** Sections of MGH-U3-derived xenograft tumours from mice treated with scramble siRNA or *TYRO3* siRNA#1 were stained for DNA fragmentation with a TUNEL assay detection kit (*n* = 4 mice per group). Representative sections are shown (left panel, black bar = 100 μm) and the percentage of cells displaying apoptosis (right panel) was evaluated. The data shown are means ± SD (*n* = 4). Statistical analysis was performed with *t*-tests

We investigated the molecular mechanisms underlying the oncogenic dependency on TYRO3 expression in bladder carcinomas, by conducting a comprehensive gene expression analysis with Affymetrix U133 Plus 2.0 arrays, in MGH-U3, RT112 and UM-UC-5 cells transfected with three different *TYRO3* siRNAs. We identified 284 genes as differentially expressed after *TYRO3* knockdown in these three cell lines (adjusted *p* value < 0.05, Ilog_2_(FC)I ≧ 0.58 (see “Methods” section); supplementary table S4). An analysis of this list of genes with Ingenuity Pathway Analysis (IPA) software identified key molecular and cellular functions (Fig. 4a, upper panel) and upstream regulators/ transcription factors presenting significant alterations upon *TYRO3* knockdown (Fig. 4a, lower panel). TYRO3-regulated genes were highly significantly enriched in gene sets involved in the “cell cycle”, “cell proliferation”, “cell survival”, “cell apoptosis” and “cell death” functions (Fig. 4a upper panel). The gene sets corresponding to each function are listed in supplementary table S5. Consistent with these findings, cell cycle and cell survival processes were significantly downregulated (negative *z*-score), whereas cell death and apoptosis, in particular, were upregulated (positive *z*-score) upon *TYRO3* knockdown (Fig. 4a, upper panel). We also identified, among the top ten transcriptional regulators whose activities were predicted to be upregulated or downregulated by *TYRO3* depletion, genes encoding important regulators of the cell cycle and cell survival such as MYC, FOXM1, E2F1, E2F2, E2F3 and CCND1, whose activity were predicted to be downregulated whereas the activities of the tumour suppressor transcription factors TP53 and RB1 were predicted to be upregulated (Fig. 4a, lower panel, table S6). Strikingly, CDKN2A was also predicted as upregulated upon TYRO3 depletion whereas cell lines harbour a CDKN2A homozygous gene deletion^21^ highlighting the need for prediction validation that can be biased if targeted genes used for a transcription regulator are also controlled by other regulators. Western blot analysis showed a downregulation of FOXM1 and CYCLIN D1 levels following *TYRO3* depletion in MGH-U3, RT112 and UM-UC-5 cells, potentially accounting for the lower levels of activity for these transcription factors highlighted by our transcriptomic data analysis in these cell lines (Fig. 4a, b, lower panel). Although IPA predicted an inhibition of *MYC*, western blot analysis showed a decrease in c-MYC protein levels mainly in UM-UC-5 and RT112. Conversely, during cell cycle progression, retinoblastoma protein (pRB) is sequentially phosphorylated by CYCLIN-CDK complexes, and CYCLIN D1-CDK4 specifically phosphorylates pRB on the Ser780 residue, leading to its inactivation and the release of E2F.^22^ In accordance with our transcriptomic data showing that both *CCND1* and *CDK4* gene expression were downregulated upon *TYRO3* depletion (supplementary table S4), we observed lower levels of pRB phosphorylation at Ser780 in the three cell lines studied after *TYRO3* knockdown, which may, therefore, lead to pRB activation and E2F inactivation, as suggested by IPA analysis of our transcriptomic data (Fig. 4a, b, lower panel). Western blot analysis also confirmed, at the protein level, the downregulation of the AURORA A and AURORA B kinases involved in cell cycle entry and mitotic spindle assembly, and of SURVIVIN, for which mRNA levels had already been shown to be decreased by *TYRO3* depletion (log_2_FC = −1.104; −1.159 and −1.298, respectively) (Fig. 4b). Taken together, our data for the MGH-U3, RT112 and UM-UC-5 cell lines demonstrate that *TYRO3* depletion inhibits proliferative pathways and activates tumour suppressor pathways. These molecular mechanisms are undoubtedly of general importance, because all our western blot results (Fig. 4b) were confirmed in two other TYRO3-dependent cell lines, VM-CUB-1 and UM-UC-9 (Supplementary Figure S7). Given that the pRB pathway is frequently altered in bladder cancer, particularly through direct *RB1* gene inactivation, we assessed the impact of *TYRO3* depletion in the *RB1* mutated bladder cancer cell lines, 5637 (pY325*), 647 V (p.Q383*) and HT1376 (p.Q383*).^17^ The cell viability of these *RB1* mutant cells was significantly decreased upon *TYRO3* silencing (Supplementary Figure S8). Although the impact of TYRO3 depletion was not as important as the one observed in *RB1* wild type cells, these results suggest that the sensitivity to TYRO3 inhibition is not strictly dependent on *RB1* gene status.

SURVIVIN is not only involved in the cell cycle, in which it regulates the mitotic spindle checkpoint, but also in the inhibition of caspase-9, an initiator caspase involved in caspase-3 activation.^23^ Its loss is, thus, consistent with the induction of apoptosis upon *TYRO3* silencing, through caspase activation (Fig. 4a, upper panel). Western blot analysis of lysates of *TYRO3* siRNA-transfected RT112 cells showed a greater accumulation, over time, of the cleaved forms of the effector caspase-3 and the initiator caspase-8, resulting in the cleavage of PARP, one of the main targets of capase-3, thereby clearly demonstrating the activation of apoptosis by *TYRO3* depletion (Fig. 4c, left panel). These results were confirmed 72 h after transfection with *TYRO3* siRNA, in MGH-U3 and UM-UC-5 cells (Fig. 4c, right panel), and cleaved PARP was also identified in VM-CUB-1 and UM-UC-9 cells after *TYRO3* knockdown (Supplementary Figure S7). Moreover, inhibitors of either caspase-3/7 (z-IEVD-fmk) or caspase-8 (z-IETD-fmk) significantly reduced the impact of *TYRO3* depletion on cell survival, demonstrating the crucial importance of apoptosis regulation for TYRO3 activity in bladder cancer cells (Fig. 4d). Finally, a TUNEL assay performed in our MGH-U3 xenograft mouse model (Fig. 3) clearly showed that apoptosis levels were significantly higher in *TYRO3* siRNA-treated animals (Fig. 4e).

## Discussion

One of the major aims of this study was to assess the expression of TAM receptors in bladder cancer. Our study showed that only TYRO3 expression levels were significantly increased in both NMIBCs and MIBCs relative to normal urothelium samples. The aberrant expression of TAM receptors is frequently observed in several cancers and has been associated with aggressive cancer phenotypes, overall poor patient survival and the emergence of drug resistance.^9^ TYRO3 has been less studied than the other two TAM receptors, but several studies have reported results similar to ours, highlighting the contribution of this receptor in various types of cancer: leiomyosarcoma, melanoma, colorectal and thyroid cancers.^18,24–27^ However, TYRO3 expression in bladder cancer is not correlated with disease stage (contrary to reports for colorectal cancer), suggesting a possible role for TYRO3 in the formation of bladder cancers, as well as in their progression. However, we can assume that TYRO3 plays a greater role in MIBCs than in NMIBCs, due to the stronger expression of its ligand, *GAS6*, in MIBCs than in NMIBCs. Nevertheless, ligand-independent activation has also been reported for strong TYRO3 expression,^6^ and we show here that TYRO3 is N-glycosylated in bladder tumours, which might lead to its ligand-independent activation in the presence of galectin, in a process similar to that described for N-glysosylated vascular endothelial growth factor receptor 2, which is activated by galectin-1 in the absence of ligand.^28^

No alteration of the *TYRO3* copy number was detected (data not shown), and the mechanisms increasing the expression of TYRO3 in bladder tumour cells remain to be determined. TYRO3 was detected at the plasma membrane in several bladder cancer cell lines by flow cytometry, but IHC staining on human bladder tumour sections showed it to be present mostly in the cytoplasm and only weakly expressed at the plasma membrane of tumour cells. A similar RTK distribution has already been reported for TYRO3 and AXL in human colorectal cancer and pancreatic cancer, respectively.^18,29^

Consistent with this general higher level of expression of *TYRO3* in bladder tumours compared to normal samples, TYRO3 expression was detected in most of the UBC cell lines, whereas AXL and MERTK had more heterogeneous expression patterns in UBC cell lines. *TYRO3* knockdown systematically resulted in a significant large decrease in cell growth/survival, in all the bladder cancer cell lines tested, regardless of AXL and MERTK expression levels, as previously reported for melanoma cells.^30^ These results suggest that neither AXL nor MERTK could play a redundant role, rescuing the loss of TYRO3. The three TAM receptors therefore have different roles in bladder tumours. Accordingly, the impact of *TYRO3* knockdown on UBC cell proliferation was consistently greater than the impact of *MERTK* or *AXL* knockdown. Despite the preponderant role of TYRO3 in the control of bladder cancer cell growth/survival, we cannot rule out the possibility that *AXL* or *MERTK* knockdown affects other processes known to be regulated by TAM receptors, such as cell migration, and that long term inhibition of AXL or MERTK could impact tumour cell viability.^31^

Our transcriptomic analysis, with validation by immunoblotting, showed that *TYRO3* silencing inhibited the cell cycle and cell survival processes, whilst inducing apoptosis. Such effects of *TYRO3* knockdown have already been reported in other cancer types: melanoma, thyroid and breast cancer cells.^25,26,32^ In breast cancer cells, as reported here for UBC cells, the regulation of these processes may involve a decrease in CYCLIN D1 and SURVIVIN levels induced by *TYRO3* knockdown.^32^ Our transcriptomic analysis suggested a role for MITF regulation, as previously reported for melanoma,^26^ but the involvement of MITF in bladder tumours has never been explored. Other pathways were identified as regulated in both studies, including proliferation, survival/growth and apoptosis, suggesting possible roles for TYRO3 in these processes and the existence of general features relating to TYRO3 activity in different cancer types. However, some cancer type-specific features may fine-tune TYRO3-mediated signalling pathways and their outcomes. Indeed, TYRO3 has been shown to stimulate not only cell proliferation, but also cell migration and EMT processes, via the regulation of *SNAI1*, in colorectal cancer,^18^ whereas our transcriptomic analysis detected no deregulation of cell migration or EMT processes following *TYRO3* knockdown in three different UBC cell lines.

In addition to this cell-autonomous effect in bladder cancer, TYRO3 may also be involved in cell-non-autonomous effects. Indeed, TAM receptors have been shown to favour the escape of tumour cells from immune surveillance by activating innate immune checkpoints and, in particular, by upregulating PD-L1 expression.^33,34^ Our transcriptomic analysis upon *TYRO3* depletion revealed no modulation of *CD274* (PD-L1) expression, but a significant increase in *TNFSF9* (CD137L) expression. Given the current interest in agonistic anti-CD137 antibodies for cancer treatment,^35^ TYRO3 inhibition might be useful for both inhibiting tumour growth and boosting the antitumour immune response. In line with this hypothesis, a phase 1b clinical trial (NCT03170960) has recently begun to test the combination of a pan-TAM inhibitor (cabozantinib) with an anti-PD-L1 antibody (atezolizumab) in patients with advanced urothelial carcinoma (including bladder, renal pelvis, ureter, or urethra carcinoma) or renal cell carcinoma.

Overall, our data suggests that TYRO3 is a promising potential therapeutic target for both muscle-invasive and non-muscle invasive bladder cancers. Although our pharmaceutical inhibition experiments with TAM inhibitors reproduced the results obtained with TAM knockdown, we cannot rule out the possibility that other activities of UNC-2025 or BMS-777607 could impact tumour cell viability. Given the preclinical evidence of the involvement of TYRO3 in cancer, it is important to develop potent small inhibitors or monoclonal antibodies specific against this tyrosine kinase receptor. Efforts in such direction are being made.^18,30^

## Supplementary information

Figure S1

Figure S2

Figure S3

Figure S4

Figure S5

Figure S6

Figure S7

Figure S8

Table S1

Table S2

Table S3

Table S4

Table S5

Table S6

## Data Availability

The microarray data are available from GEO (https://www.ncbi.nlm.nih.gov/geo/) under accession number GSE100025.
