# Supplementary figures and images for: TYRO3 as a molecular target for growth inhibition and apoptosis induction in bladder cancer
Source: Br J Cancer. 2019 Feb 15;120(5):555–64. doi: 10.1038/s41416-019-0397-6 (PMC6461973; doi:10.1038/s41416-019-0397-6)

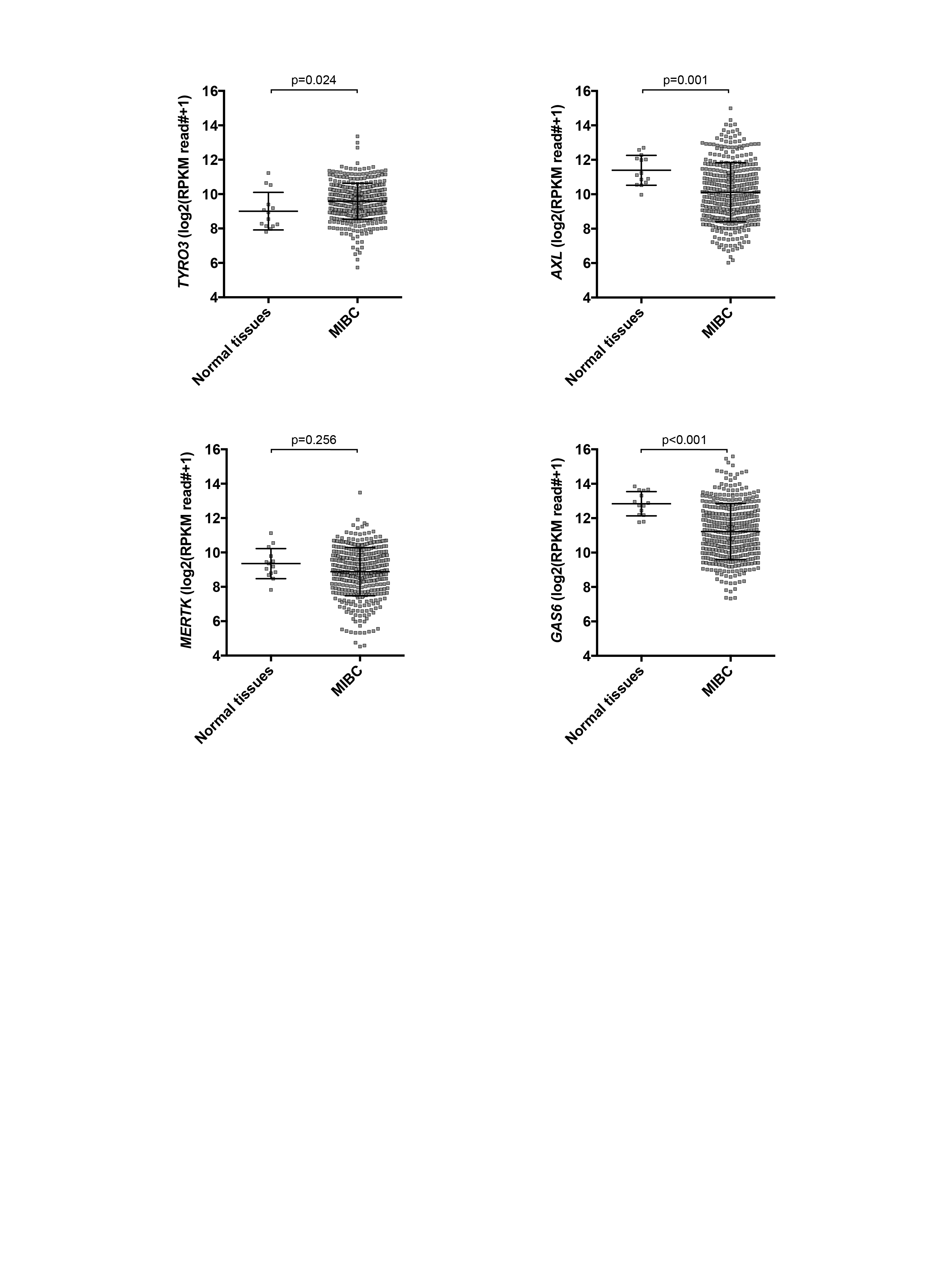

Supplement: Supplementary file 1 — Figure S1 [file 41416_2019_397_MOESM1_ESM.tif]

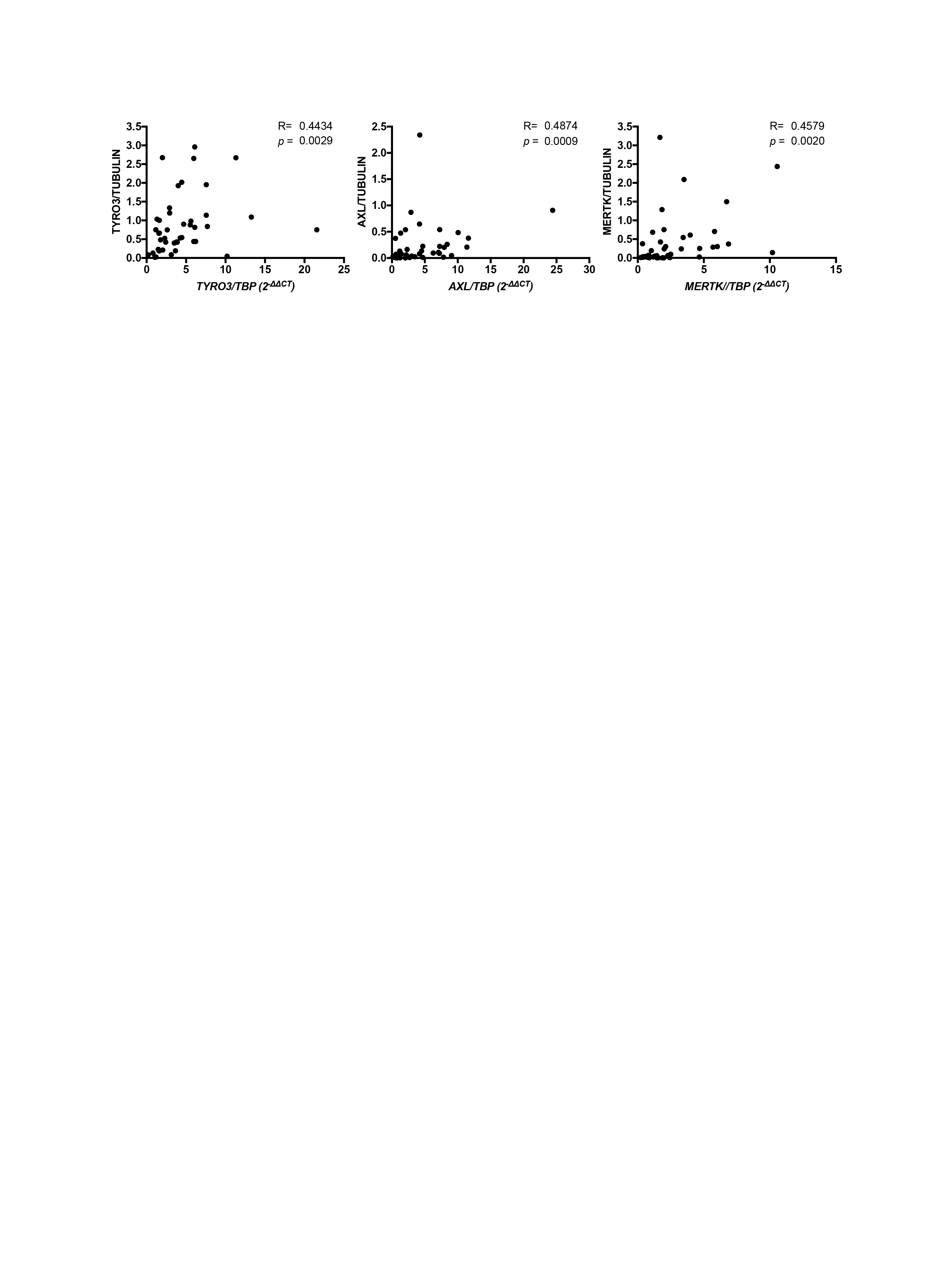

Supplement: Supplementary file 2 — Figure S2 [file 41416_2019_397_MOESM2_ESM.tif]

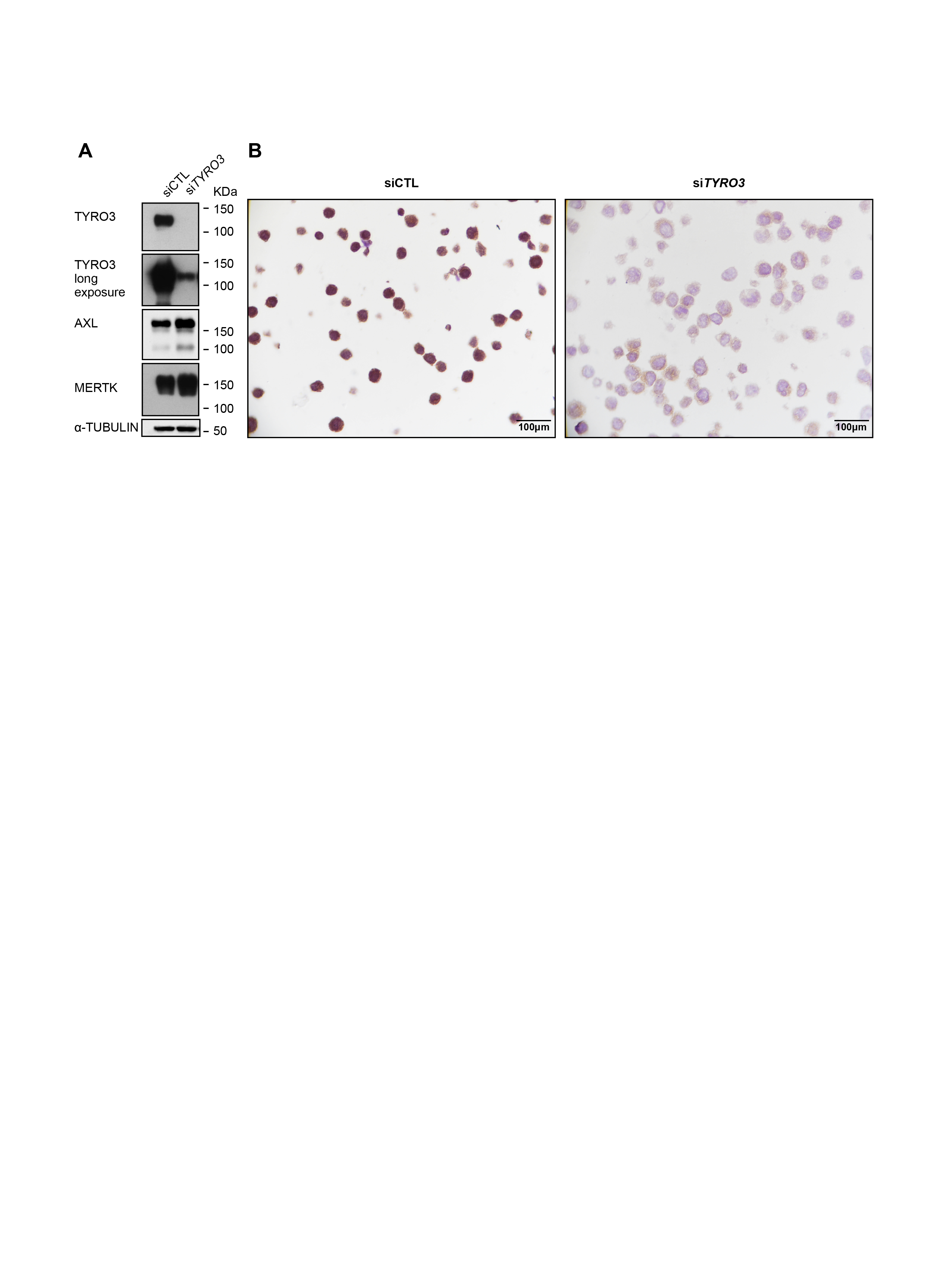

Supplement: Supplementary file 3 — Figure S3 [file 41416_2019_397_MOESM3_ESM.tif]

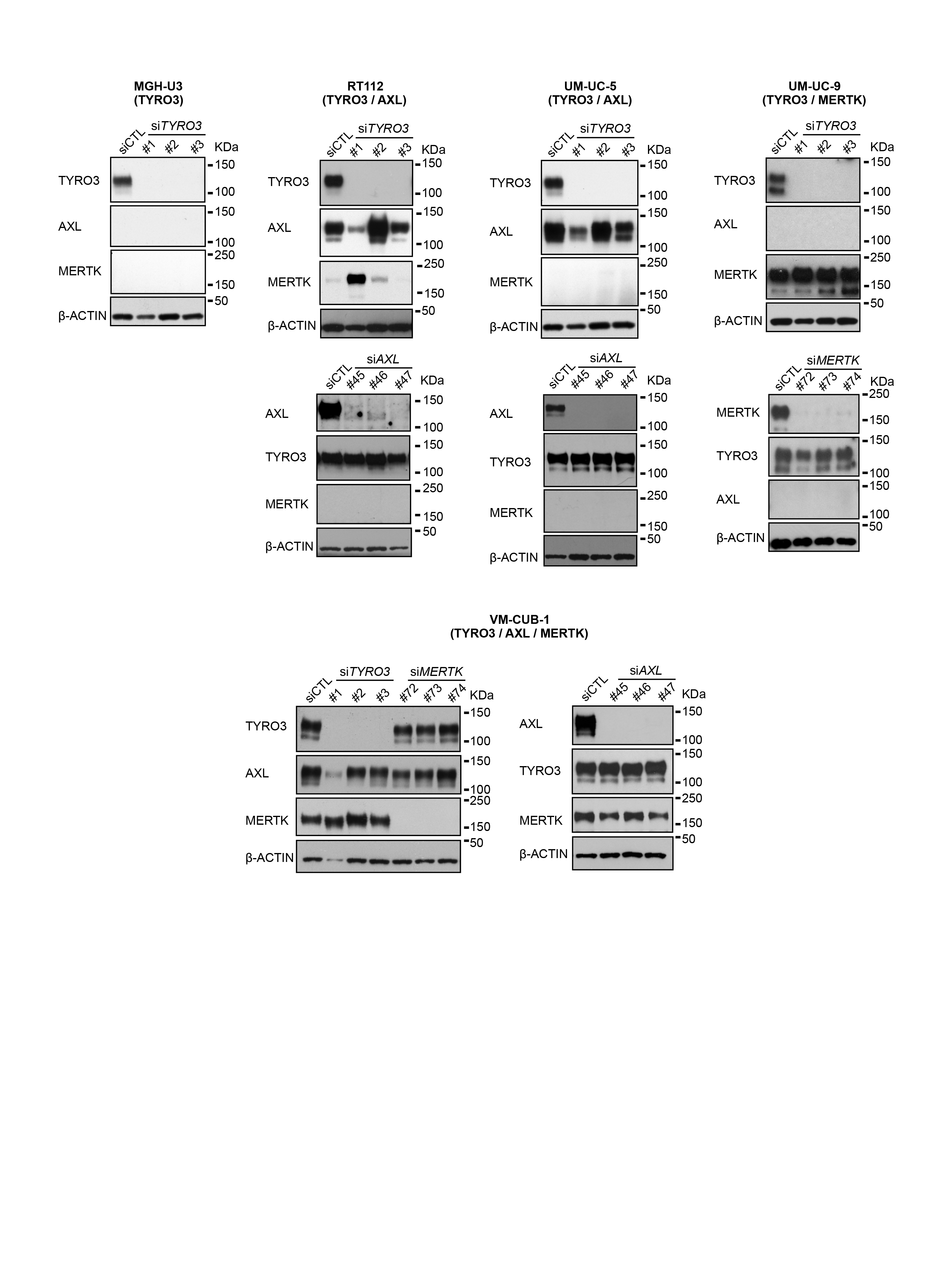

Supplement: Supplementary file 4 — Figure S4 [file 41416_2019_397_MOESM4_ESM.tif]

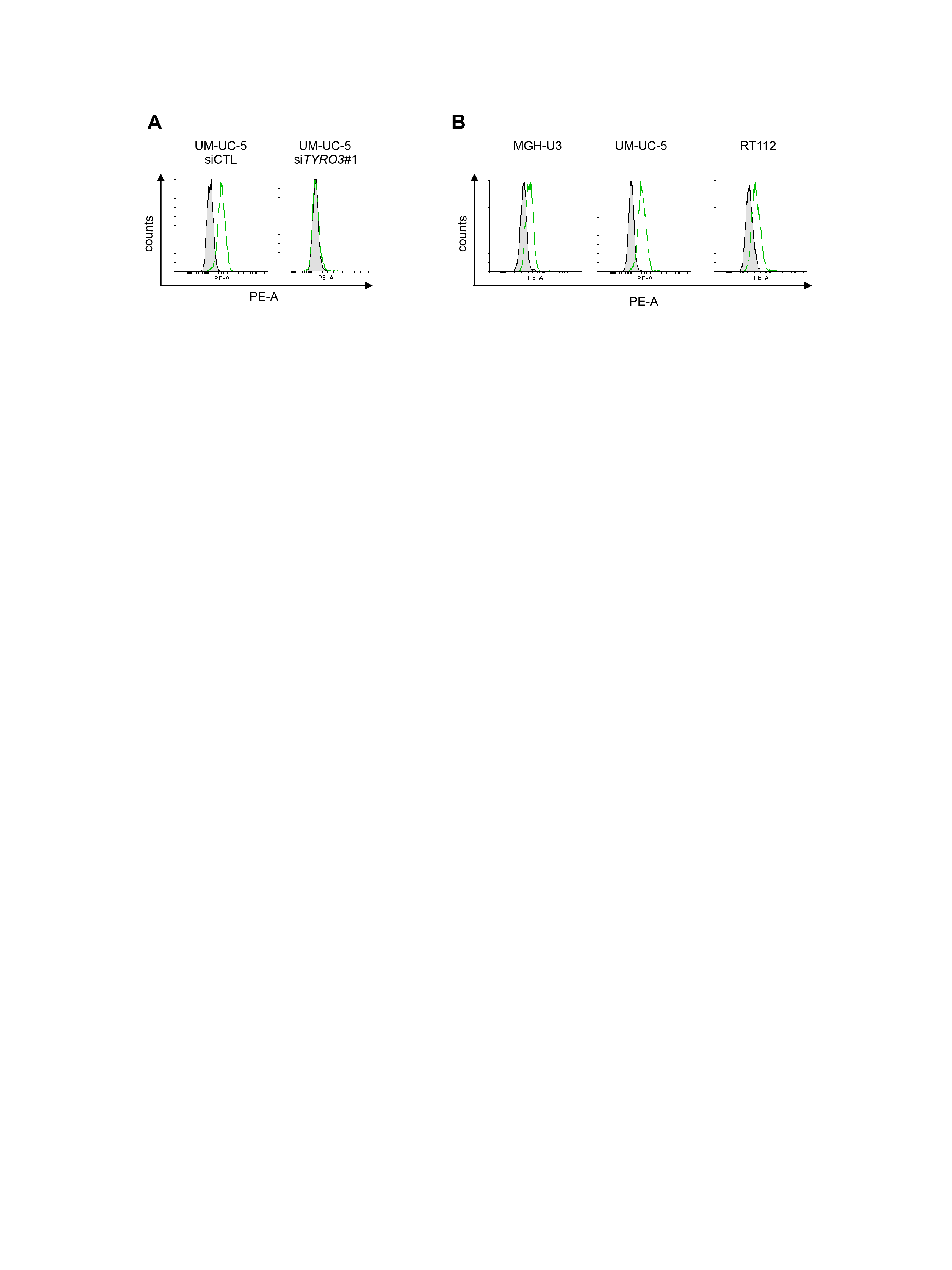

Supplement: Supplementary file 5 — Figure S5 [file 41416_2019_397_MOESM5_ESM.tif]

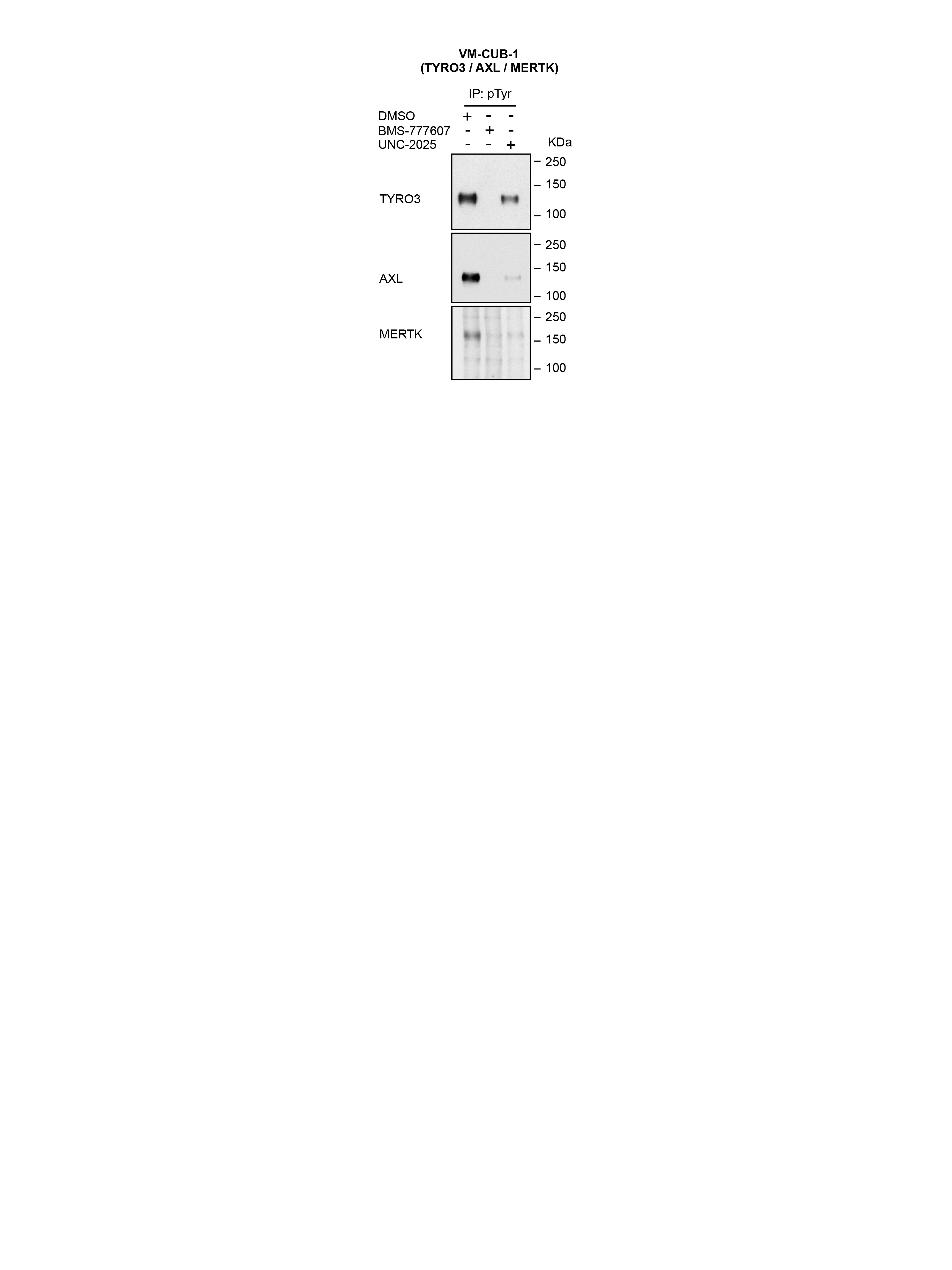

Supplement: Supplementary file 6 — Figure S6 [file 41416_2019_397_MOESM6_ESM.tif]

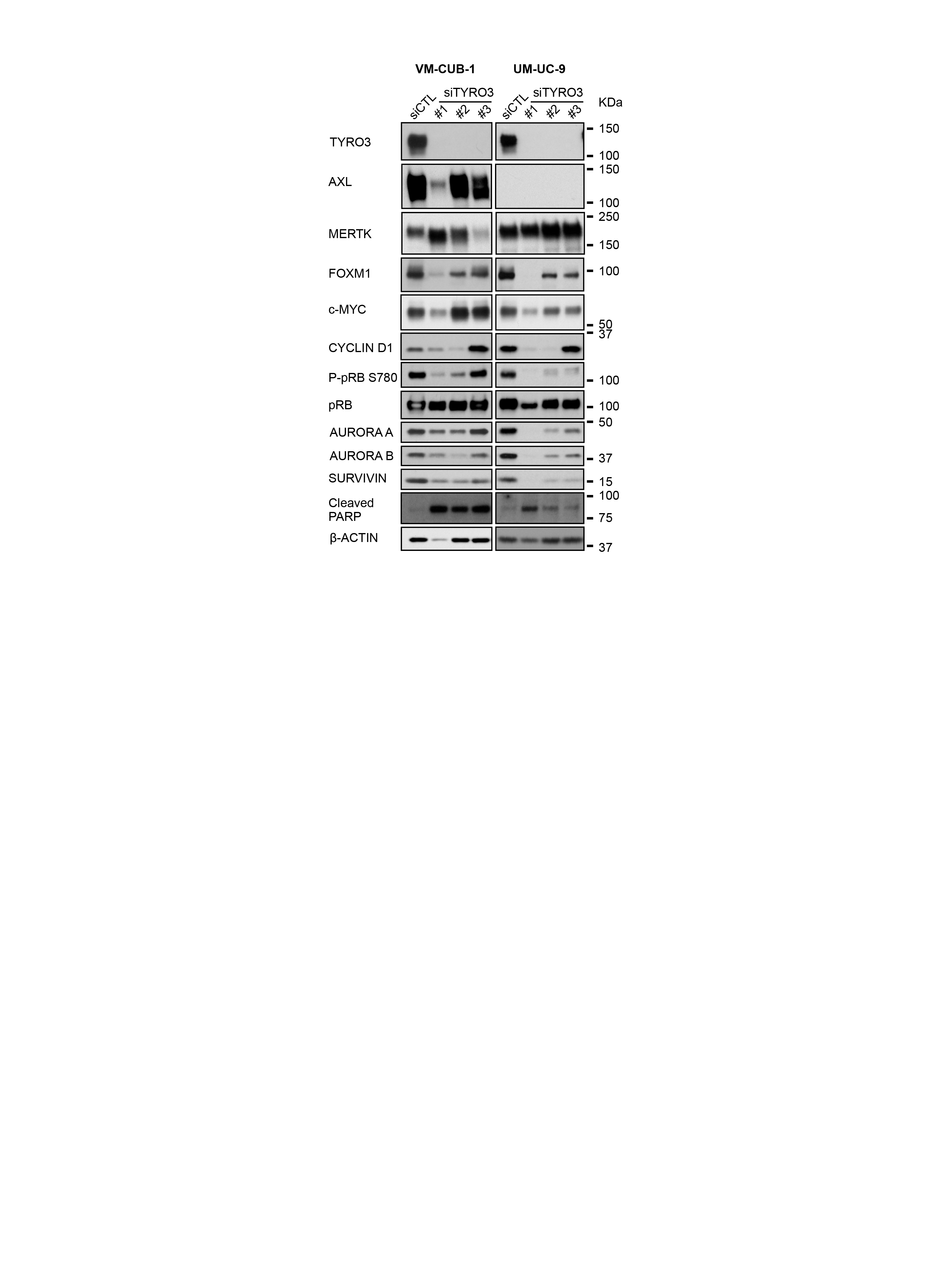

Supplement: Supplementary file 7 — Figure S7 [file 41416_2019_397_MOESM7_ESM.tif]

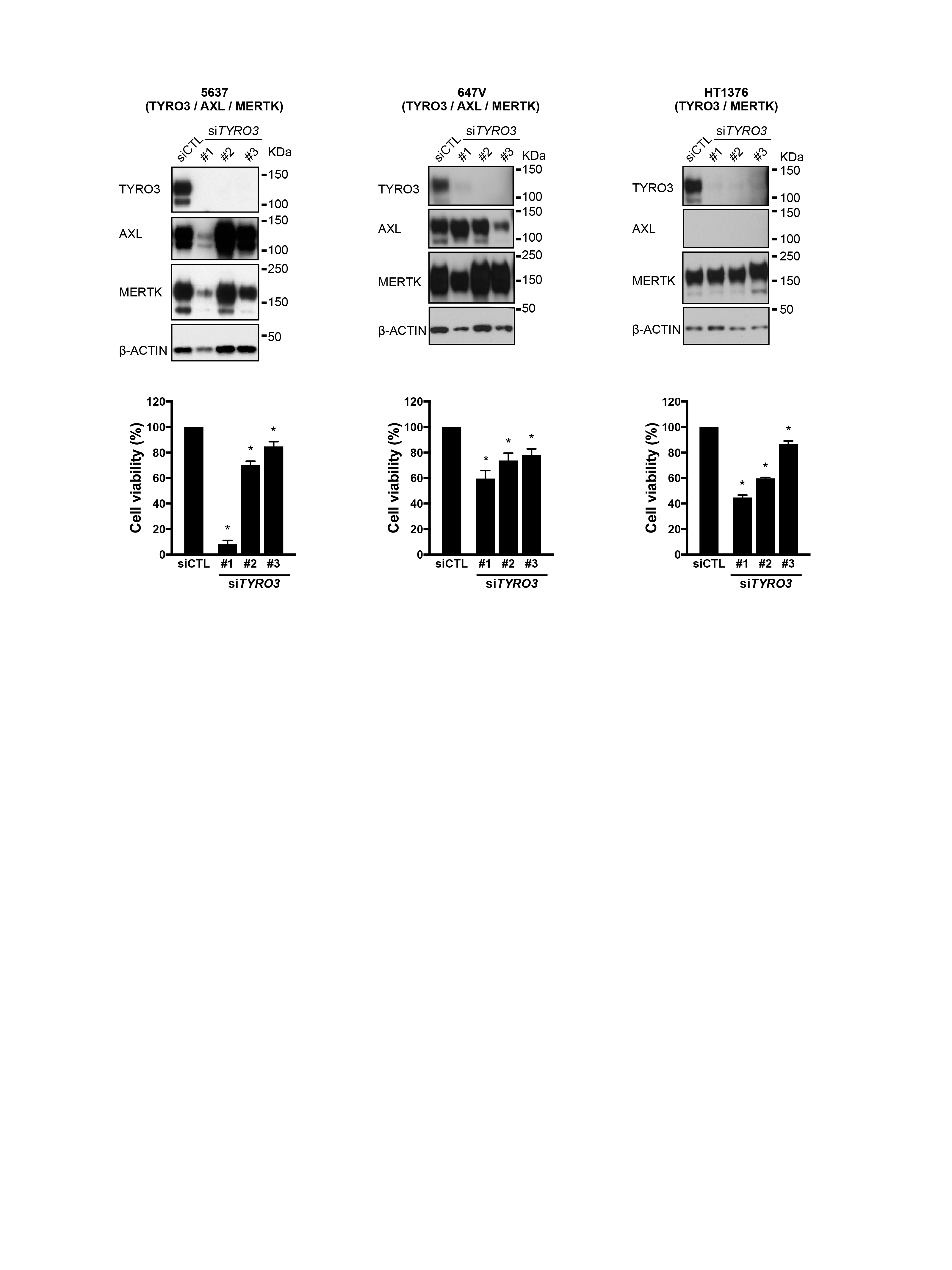

Supplement: Supplementary file 8 — Figure S8 [file 41416_2019_397_MOESM8_ESM.tif]
